# Antitumor activity of phenethyl isothiocyanate in HER2-positive breast cancer models

**DOI:** 10.1186/1741-7015-10-80

**Published:** 2012-07-24

**Authors:** Parul Gupta, Sanjay K Srivastava

**Affiliations:** 1 Department of Biomedical Sciences and Cancer Biology Center, Texas Tech University Health Sciences Center, Amarillo, TX, USA

**Keywords:** apoptosis, doxorubicin, EGFR, ERBB2/HER2, *in vivo*, mitochondria, STAT3

## Abstract

**Background:**

HER2 is an oncogene, expression of which leads to poor prognosis in 30% of breast cancer patients. Although trastuzumab is apparently an effective therapy against HER2-positive tumors, its systemic toxicity and resistance in the majority of patients restricts its applicability. In this study we evaluated the effects of phenethyl isothiocyanate (PEITC) in HER2-positive breast cancer cells.

**Methods:**

MDA-MB-231 and MCF-7 breast cancer cells stably transfected with HER2 (high HER2 (HH)) were used in this study. The effect of PEITC was evaluated using cytotoxicity and apoptosis assay in these syngeneic cells. Western blotting was used to delineate HER2 signaling. SCID/NOD mice were implanted with MDA-MB-231 (HH) xenografts.

**Results:**

Our results show that treatment of MDA-MB-231 and MCF-7 cells with varying concentrations of PEITC for 24 h extensively reduced the survival of the cells with a 50% inhibitory concentration (IC_50_) of 8 μM in MDA-MB-231 and 14 μM in MCF-7 cells. PEITC treatment substantially decreased the expression of HER2, epidermal growth factor receptor (EGFR) and phosphorylation of signal transducer and activator of transcription 3 (STAT3) at Tyr-705. The expression of BCL-2-associated × (BAX) and BIM proteins were increased, whereas the levels of B cell lymphoma-extra large (BCL-XL) and X-linked inhibitor of apoptosis protein (XIAP) were significantly decreased in both the cell lines in response to PEITC treatment. Substantial cleavage of caspase 3 and poly-ADP ribose polymerase (PARP) were associated with PEITC-mediated apoptosis in MDA-MB-231 and MCF-7 cells. Notably, transient silencing of HER2 decreased and overexpressing HER2 increased the effects of PEITC. Furthermore, reactive oxygen species (ROS) generation, mitochondrial depolarization and apoptosis by PEITC treatment were much higher in breast cancer cells expressing higher levels of HER2 (HH) as compared to parent cell lines. The IC_50 _of PEITC following 24 h of treatment was reduced remarkably to 5 μM in MDA-MB-231 (HH) and 4 μM in MCF-7 (HH) cells, stably overexpressing HER2. Oral administration of 12 μM PEITC significantly suppressed the growth of breast tumor xenografts in SCID/NOD mice. In agreement with our *in vitro *results, tumors from PEITC-treated mice demonstrated reduced HER2, EGFR and STAT3 expression and increased apoptosis as revealed by cleavage of caspase 3 and PARP. In addition our results show that PEITC can enhance the efficacy of doxorubicin.

**Conclusions:**

Our results show a unique specificity of PEITC in inducing apoptosis in HER2-expressing tumor cells *in vitro *and *in vivo *and enhancing the effects of doxorubicin. This unique specificity of PEITC offers promise to a subset of breast cancer patients overexpressing HER2.

## Background

The HER2/neu protein belongs to the family of epidermal growth factor receptors (EGFRs) and is known to be amplified in several neoplasms such as breast, salivary gland, stomach, kidney and lung. It is overexpressed in about 30% of breast cancer patients [1-6]. The cytoplasmic domain of this intact tyrosine kinase receptor has been implied to generate normal mitogenic as well as transforming signals [7], indicating its direct role in cell proliferation [8]. HER2 expression has been correlated with poor prognosis in cancer patients by mechanisms such as impaired DNA repair [9], angiogenesis [10] and metastasis [11]. The oncogenic property of HER2 can be well enunciated, but its role in chemotherapy is not clearly elucidated because of its obscured molecular mechanisms. Though there are effective approaches for targeting HER2 such as trastuzumab (herceptin), which is a specific antibody for HER2, it is associated with toxicity and/or resistance. This implies a need for the search of better therapeutic agents that can target HER2-positive cancer cells.

Doxorubicin (Adriamycin) is used to treat patients in the early stages of breast cancer. The normal dosage of doxorubicin usually ranges 40 to 60 mg/m^2^. It is well established that a significant percentage of patients on doxorubicin therapy face a risk of cardiotoxocity at these doses [12]. A lifetime cumulative dose of about 500 mg/m^2 ^has been associated with chronic cardiotoxicity [13]. To avoid the drawbacks of doxorubicin, it is important to find novel therapies that can reduce its dose without compromising its therapeutic effects. It has been shown that doxorubicin can downregulate EGFR but not HER2 [14]. The approach of combination therapies can help to enhance its efficacy in HER2-positive patients.

Interestingly in two clinical studies, it was observed that HER2-positive tumors responded better to the chemotherapy regimen than HER2-negative tumors [15,16]. Another study illustrated that cleavage of HER2 mediated apoptosis in cancer cells by inducing intrinsic apoptosis pathway [17]. These studies indicated that the role of HER2 may be reciprocating between antiapoptotic and proapoptotic, but the conditions and agents that modulated this balance need to be identified. These studies also raised a question on the role of HER2 in cancer and provide rationale to study its possible dual role.

Various recent epidemiological studies have indicated that consumption of cruciferous vegetables such as garden cress, broccoli and so on, reduces the risk of breast cancer [18,19]. The glucosinolate-derived phenethyl isothiocyanates (PEITC) present in these vegetables has promising antitumorigenic effects, as mentioned by various studies [19-23]. Clinical studies on PEITC for lung cancer are currently underway [24]. These facts reinforce the importance and necessity of more preclinical studies required on this compound to expand and discover its true potential in breast cancer.

Our current studies establish the anticancer effects of PEITC in HER2-positive tumor cells. Our results show that the growth suppressive effects of PEITC in breast cancer cells were mediated by HER2 depletion in both *in vitro *and *in vivo *models and that HER2-expressing cells were more sensitive to PEITC-induced apoptosis. In addition, our study shows that in combination, PEITC can significantly enhance the apoptosis-inducing effects of doxorubicin. Our study provides a direction towards the mechanism of HER2-mediated sensitivity of cells to chemotherapy.

## Methods

### Cell culture

Human breast carcinoma cell lines MCF-7 and MDA-MB-231 were obtained from American Type Culture Collection (ATCC; http://www.atcc.org) and were maintained in minimal essential medium (MEM) supplemented with 10% fetal bovine serum (FBS), 5% penicillin/streptomycin/neomycin (PSN) and 0.01 mg/ml insulin and Dulbecco's modified Eagle medium (DMEM) supplemented with 10% FBS and 5% PSN, respectively, unless otherwise stated. The HER2 overexpressing cells MDA-MB-231 (high HER2 (HH)) cells and their vector controls were kindly provided by Dr Patricia S Steeg (National Institutes of Health, Bethesda, MD, USA) and Dr Quentin Smith (Texas Tech University Health Sciences Center, Amarillo, TX, USA) and were maintained in DMEM supplemented with 10% FBS, 5% penicillin, streptomycin, neomycin solution and 300 μg/ml zeocin. HER2 was transfected in brain seeking subset of MDA-MB-231 cells. No significant difference was observed at molecular level in these cells and parental MDA-MB-231 cells. The MCF-7 overexpressing HER2 cells were kindly provided by Dr Huang Fei (Bristol-Myers Squibb Co., Princeton, NJ, USA) and were cultured in DMEM supplemented with 10% FBS and 5% PSN. The ATCC uses DNA fingerprinting (microsatellite analysis) for cell line authentication. All the cells used in this study were within 20 passages after receipt or resuscitation.

### Cytotoxicity studies

Cells were plated at a density of 2,000 to 5,000 cells/well in 96-well plates, allowed to attach overnight and treated with different concentrations of PEITC (molecular weight 163.2; purity > 99%; Sigma Aldrich, St. Louis, MO, USA) for various time intervals. The cells were fixed with ice cold 10% trichloroacetic acid, washed and stained with sulforhodamine B dye. The optical density was measured in Tris base solution using a plate reader after washing the dye with 1% acetic acid solution as described by us previously [25].

### Annexin V-fluorescein isothiocyanate (FITC) apoptosis assay

The apoptosis assay was performed using a kit (BD Biosciences, San Jose, CA, USA) according to manufacturer's instructions. Approximately 0.3 × 10^6 ^cells were plated into six-well plates and left overnight for attachment. After treatment for 24 h with 0 to 15 μM PEITC, cells were harvested by trypsinization and suspended in phosphate-buffered saline (PBS) to a cell density of 1 × 10^6^/ml. A total of 100 μl of binding buffer, 5.0 μl of Annexin V-FITC and 5 μl of propidium iodide were added to the suspension and incubated for additional 20 minutes at room temperature in the dark. Total sample volume was made up to 500 μl with binding buffer. Samples were incubated on ice in the dark and analyzed by flow cytometer after vortexing (Accuri C6, Ann Arbor, MI, USA).

### Enzyme-linked immunosorbent assay (ELISA) apoptosis assay

Histone associated DNA fragmentation during apoptosis was analyzed using an ELISA kit (Roche Applied Science, Indianapolis, IN, USA). Approximately 1.0 × 10^4 ^cells were plated in each well in a 96 well plate and left overnight for attachment. After being treated with different concentrations of PEITC for 24 h, cells were processed according to the manufacturer's instructions and as described by us previously [26].

### Hydrogen peroxide determination

Cells were plated in a six-well plate at a density of 0.3 × 10^6 ^cells per well and were allowed to attach overnight. After 24 h of treatment with PEITC, cells were harvested and lysed as described under western blotting analysis to collect and estimate the protein. The protein was transferred to 96 well plates at a concentration of 5 μg per well. The hydrogen peroxide (H_2_O_2_) content was determined using the Quantichrom (Bioassay Systems, Hayward, CA, USA) peroxide assay kit as per the manufacturer's instructions and described by us [27].

### Mitochondrial membrane potential

Cells were plated at a density of 0.3 × 10^6 ^cells/well in a six-well plate, allowed to attach overnight and treated with 10 μM PEITC for 24 h. Prior to collection, cells were incubated with 100 nM tetramethylrhodamine (TMRM) (Life Technologies, Grand Island, NY, USA) for 20 minutes. Cells were washed with PBS, resuspended in fluorescence-activated cell sorting (FACS) running buffer and analyzed by flow cytometry (Accuri C6) as described by us [27].

### Western blot analysis

The cells were treated with varying concentrations of PEITC (5, 10 and 15 μM) for 24 h. In a time-dependent study, cells were treated with 10 μM PEITC for 0, 1, 2, 4, 8, 12, 16, 24 and 48 h. In another experiment, MDA-MB-231 cells were treated with 10 mM Tiron for 1 h prior to treatment with 10 μM PEITC for 24 h. Whole cell lysates were prepared using 4% (w/v) CHAPS buffer (6 M urea, 2 M thiourea, 10 mM Tris pH 7.4) while tumor lysates were prepared by homogenizing the tumors in PBS. Control and treated protein was subjected to sodium dodecyl sulfate polyacrylamide gel electrophoresis (SDS-PAGE), and the segregated protein was transferred on a polyvinylidene fluoride (PVDF) membrane. The membrane was developed as described by us previously using primary antibodies specific to human HER2 (Abcam, Cambridge, MA, USA; 1:700), EGFR (Cell Signaling, Danvers, MA, USA; 1:1000), phosphorylated signal transducer and activator of transcription 3 (p-STAT3) (Y705) (Cell Signaling, Danvers, MA, USA; 1:1000), STAT3 (Cell Signaling, Danvers, MA, USA; 1:1000), BIM (Cell Signaling, Danvers, MA, USA; 1:2000), BCL-2-associated × (BAX) (Cell Signaling, Danvers, MA, USA; 1:2000), B-cell lymphoma-extra large (BCL-XL) (Cell Signaling, Danvers, MA, USA; 1:1000), X-linked inhibitor of apoptosis protein (XIAP) (Cell Signaling, Danvers, MA, USA; 1:1000), cleaved caspase 3 (Cell Signaling, Danvers, MA, USA; 1:1000), cleaved poly-ADP ribose polymerase (PARP) (Cell Signaling, Danvers, MA, USA; 1:1000) and actin (Sigma Aldrich, St. Loius, MO, USA; 1:10,000) [28,29].

### Mitochondria-free cytosolic fractionation

The cell fractionation was performed using the mitochondrial extraction kit (Pierce, Rockford, IL, USA) as per the manufacturer's instructions. The cells were plated at density of 5 × 10^6 ^cells per 150 mm petri dish, left at incubation for attachment overnight and then treated with 10 μM PEITC for 24 h. Cells were harvested using trypsin after treatment and processed as per manufacturer's instructions.

### HER2 silencing using HER2 small interfering (si)RNA

Approximately 0.3 × 10^6 ^cells were plated in a six-well plate and left overnight for attachment. The next day cells were transfected with HER2 siRNA or scrambled siRNA (Cell Signaling, Danvers, MA, USA) as per manufacturer's instructions using siPORT transfection reagent. After 2 days of transfection, cells were treated with 10 μM PEITC for 24 h, and the cell lysate was used for western blotting.

### HER2 overexpression by transient transfection

HER2 plasmid with pcDNA3 vector backbone was obtained from Addgene. Approximately 0.3 × 10^6 ^cells per well were plated in six-well plates and left overnight for attachment. The next day cells were transfected with 5 μg HER2 plasmid using Xfect Transfection reagent (Clontech, Mountainview, CA, USA) as per manufacturer's instructions. After 2 days of transfection, cells were treated with 10 μM PEITC. After 24 h of treatment, cell lysate was analyzed by western blotting.

### Immunofluorescence

Sections of approximately 20 micron were obtained from snap frozen tumor tissues for tumor analysis. The sections were gently placed on positively charged slides, fixed using 4% paraformaldehyde and blocked with 5% goat serum for 60 minutes as described by us previously [30]. For immunostaining cells were plated in a six-well plate on a coverslip at a density of 0.1 × 10^6 ^cells/well, allowed to attach overnight and treated with 10 μM PEITC for 24 h. Cells were rinsed twice with PBS for 5 minutes each, fixed with an ice cold mixture of acetone and methanol (1:1), rinsed with PBS and blocked with 5% goat serum for 60 minutes. Eventually, cells and tumor sections were probed with antibodies specific to HER2, p-STAT3 (Y705) and cleaved caspase 3 overnight at 4°C, followed by incubation with Alexa Fluor 488 (anti-mouse) and Alexa Fluor 594 (anti-rabbit) secondary antibodies for 1 h by gentle rocking at room temperature. After washing the cells with PBS three times, the nucleus was counterstained with DRAQ 5 (Axxora LLC, San Diego, CA, USA). Cells and tumor sections were photographed under microscope (Olympus Inc.) after coverslips were mounted on the slides.

### Tumor therapy model

Female SCID/NOD mice (4 to 6 weeks old) were obtained from Charles River (Wilmington, MA, USA) and maintained under specific pathogen-free conditions. The use of SCID/NOD mice and their treatment was approved by the Institutional Animal Care and Use Committee (IACUC), Texas Tech University Health Sciences Center, and the experiments were conducted in strict compliance with the regulations. Mice were given antioxidant-free AIN-76A special diet (TestDiet, Richmond, IN, USA) 1 week before starting the experiment. Exponentially growing MDA-MB-231 (HH) cells were harvested, washed twice with PBS and resuspended in 1:1 PBS/matrigel at a density of 50 × 10^6 ^cells per ml. A suspension of 0.1 ml containing 5 × 10^6 ^cells was injected in the right flank subcutaneously in the recipient mouse. Tumor volumes were measured three times a week (Monday, Wednesday and Friday), and animal weights were taken twice a week (Monday and Friday) as described by us previously [26,30]. The tumor volumes were calculated from tumor lengths and widths using the formula (length × (width)^2^/2) [31]. When the tumors reached a size of about 150 mm^3^, mice were randomly segregated into two groups with ten mice in each group. Test group of mice received 12 μmol PEITC (81 mg/kg) in PBS by oral gavage every day until day 35, whereas control mice received vehicle alone. No obvious signs of toxicity or discomfort were observed in the mice orally gavaged over the time. Mice were killed on day 35 by CO_2 _overdose, and death was confirmed by cervical dislocation in accordance with IACUC guidelines. The tumors were removed aseptically from each mouse and were snap frozen in liquid-nitrogen after taking the tumor weights for western blot analysis and immunofluorescence analysis.

### Combination of doxorubicin and PEITC

The effect of PEITC in combination with conventional chemotherapy agent doxorubicin (purity > 98%, Sigma Aldrich, St. Loius, MO, USA) was evaluated in MDA-MB-231 and MDA-MB-231 (HH) cells. In brief, cells were treated with 4 μM PEITC and 3 μM doxorubicin for 24 h and analyzed by sulforhodamine B cell survival assay and western blotting as described above.

### Statistical analysis

The statistical calculations and analysis were performed using Prism 5.0 (GraphPad Software Inc., San Diego, CA, USA). Results represent means ± SD or SEM of at least three independent experiments. Data was analyzed by Student's t test. Differences were considered statistically significant at *P *< 0.05.

## Results

### PEITC inhibits the proliferation of breast cancer cells

**Figure 1:**
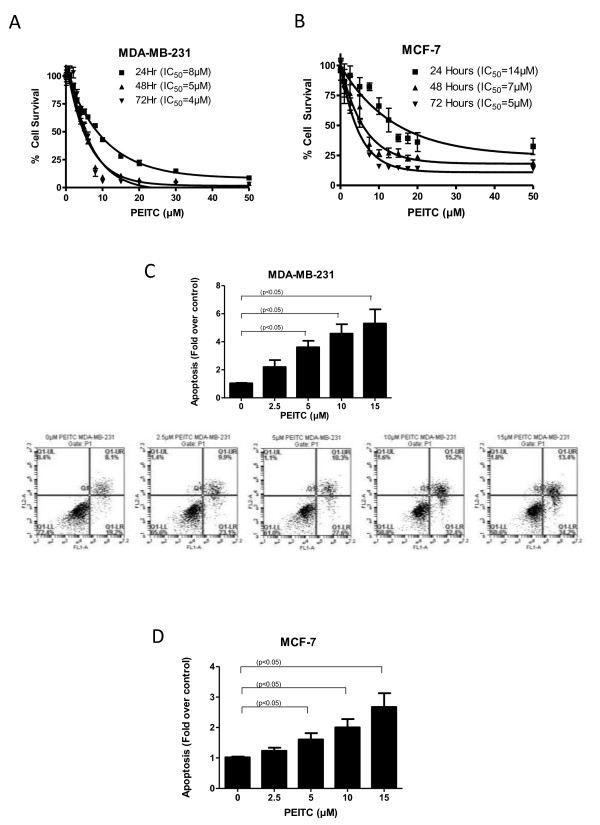
**Phenethyl isothiocyanate (PEITC) induces cell death in breast cancer cells**. **(A) **MDA-MB-231, **(B) **MCF-7 cells were treated with increasing concentrations of PEITC for 24, 48 and 72 h. Cell survival was measured with sulforhodamine B assay to estimate the IC_50 _values. The experiments were repeated at least three times with eight replicates in each experiment. **(C) **MDA-MB-231 and **(D) **MCF-7 cells were treated with or without PEITC (2.5, 5, 10, 15 μM) for 24 h. Cells were then collected and labeled with annexin V/fluorescein isothiocyanate (FITC). Cells that were positive for annexin or propidium iodide or both were measured using flow cytometry. Representative images are shown. Each experiment was repeated more than three times independently. *Statistically different when compared with control (*P *< 0.05).

We first determined the optimum concentration at which PEITC could inhibit the proliferation of breast cancer cells. Treatment of MDA-MB-231 cells with increasing concentrations of PEITC significantly reduced the survival of these cells in a concentration and time-dependent manner with a 50% inhibitory concentration (IC_50_) of 8 μM and 4 μM following 24 and 72 h of treatment respectively (Figure 1A). However, the effect of PEITC in MCF-7 cells was relatively less under similar conditions with an IC_50 _of 14 μM and 5 μM after 24 and 72 h of treatment respectively (Figure 1B), indicating that MCF-7 cells were relatively resistant to the cytotoxic effects of PEITC as compared to MDA-MB-231 cells.

### PEITC causes induction of apoptosis

To gain further insight into the mechanism of the growth inhibitory effects of PEITC, MDA-MB-231 and MCF-7 cells were treated with different concentrations of PEITC for 24 h and analyzed for apoptosis using Annexin V assay. As shown in Figure 1C, PEITC treatment induced about fourfold apoptosis in MDA-MB-231 cells at concentration as low as 5 μM. However, 5 μM PEITC induced twofold apoptosis in MCF-7 cells (Figure 1D). To confirm these observations, apoptosis was also evaluated using a cell death detection ELISA kit. Consistent with our Annexin V apoptosis data, PEITC induced apoptosis in both MDA-MB-231 and MCF-7 cells in a concentration-dependent manner (Additional file 1, Figure S1A, B).

### PEITC treatment depletes the HER2 level and induces the mitochondrial pathway of apoptosis

**Figure 2:**
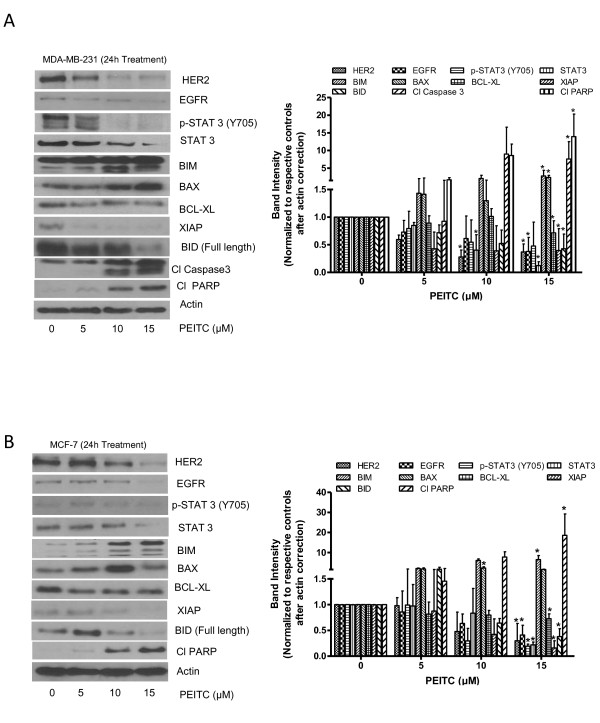
**Phenethyl isothiocyanate (PEITC) downregulates HER2 signaling in breast cancer cells**. **(A) **MDA-MB-231 and **(B) **MCF-7 cells were treated with varying concentrations of PEITC for 24 h. Representative blots showing the concentration dependent effect of PEITC on HER2, epidermal growth factor receptor (EGFR), phosphorylated signal transducer and activator of transcription 3 (p-STAT3) (Y705), BIM, BCL-2-associated × (BAX), B-cell lymphoma-extra large (BCL-XL), X-linked inhibitor of apoptosis protein (XIAP), BH3 interacting-domain death agonist (BID), cleaved caspase 3 and cleaved poly-ADP ribose polymerase (PARP). Actin was used as internal loading control. Each experiment was repeated three times independently.

**Figure 3:**
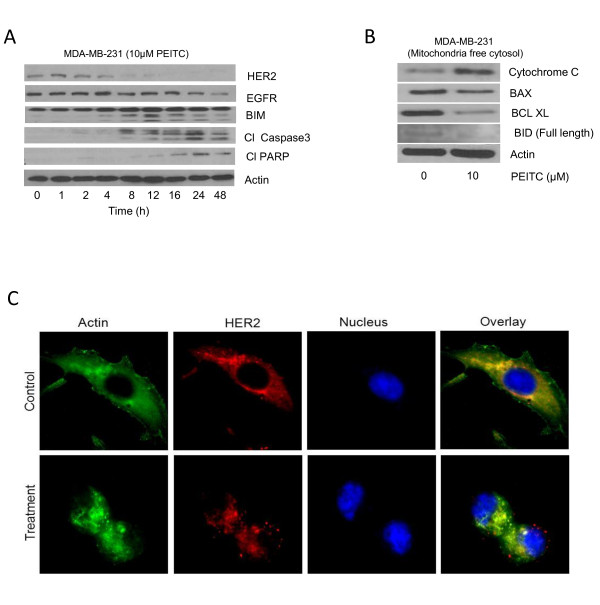
**HER2 downregulation by phenethyl isothiocyanate (PEITC) leads to mitochondrial death pathway**. **(A) **Time-dependent effect of 10 μM PEITC in MDA-MB-231 cells. **(B) **PEITC treatment mediated cytochrome c release in the cytosol. Mitochondria-free cytosol was used to detect cytochrome c in control and treated cells. Actin was used as loading control. **(C) **Representative images of HER2 immunofluorescence in MDA-MB-231 in control and PEITC treated cells. Each experiment was repeated three times independently.

To determine the mechanism of cell death induced by PEITC treatment, cell lysates were analyzed by western blotting. Consistent with previous observations MDA-MB-231 and MCF-7 cells showed constitutive levels of HER2 (Figure 2A, B) [32-34]. Our results show that treatment of MDA-MB-231 cells with PEITC after 24 h of treatment significantly reduced HER2 and EGFR expression in a concentration-dependent manner (Figure 2A). PEITC treatment also substantially reduced the expression and phosphorylation of STAT3 at Tyr-705. STAT3 plays a critical role in the expression of cell proliferative pathways and is known to be activated in breast tumors [35]. Furthermore, treatment of cells with PEITC notably increased the levels of proapoptotic proteins such as BIM and BAX and concomitantly decreased the expression of antiapoptotic proteins such as XIAP and BCL-XL (Figure 2A). We also observed an increase in the cleaved fragments of caspase 3 and PARP in PEITC-treated MDA-MB-231 cells, indicating apoptosis (Figure 2A). Similar observations were made in the other breast cancer cell line MCF-7 (Figure 2B). In a time-dependent experiment, HER2 downregulation started around 8 h of PEITC treatment in MDA-MB-231 cells (Figure 3A). Interestingly, cleavage of proapoptotic proteins BIM and caspase 3 started appearing around 8 h of PEITC treatment, suggesting that HER2 downregulation was associated with the induction of apoptosis (Figure 3A). Since PEITC-modulated BAX and BH3 interacting-domain death agonist (BID) expression indicated the involvement of mitochondria, induction of apoptosis by mitochondrial pathway was further confirmed by the release of cytochrome c into cytosol (Figure 3B). As shown in Figure 3B, 10 μM PEITC treatment for 24 h substantially increased cytosolic cytochrome c level and decreased the expression of BCL-XL. The downregulation of HER2 was also confirmed by the immunofluorescence. The staining of HER2 was markedly less in PEITC treated MDA-MB-231 cells as compared to control cells (Figure 3C).

### HER2 silencing blocks PEITC-induced apoptosis

**Figure 4:**
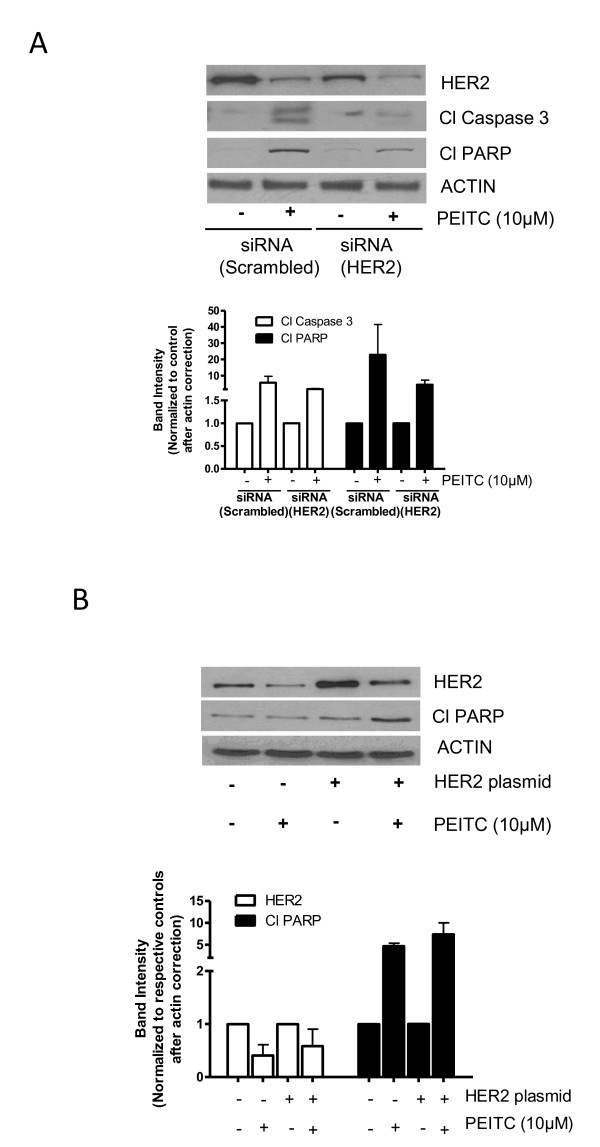
**Change in HER2 expression modulates the effect of (PEITC)**. **(A) **Effect of PEITC in HER2-silenced MDA-MB-231 cells. At 48 h after transfection of cells with HER2 siRNA, cells were treated with or without 10 μM PEITC for 24 h. Whole cell lysate was evaluated for phosphorylation of signal transducer and activator of transcription 3 (STAT3), cleavage of caspase 3 and poly-ADP ribose polymerase (PARP). **(B) **Effect of transient HER2 overexpression on apoptosis induction in MCF-7 by PEITC treatment. After 48 h of HER2 transfection, cells were treated with or without 10 μM PEITC for 24 h. The means of two independent experiments performed in duplicate are shown.

To evaluate the role of HER2 in PEITC-induced apoptosis, HER2 was silenced in MDA-MB-231 cells using HER2 siRNA. We were able to silence about 78% of HER2 expression by HER2 siRNA in MDA-MB-231 cells (Figure 4A). The silencing of HER2 reduced the cleavage of caspase 3 and PARP by PEITC treatment as compared to control MDA-MB-231 cells transfected with scrambled siRNA (Figure 4A). These results were also confirmed by the ELISA cell death detection assay (Additional file 2, Figure S2A). Silencing of HER2 reduced cell death induced by PEITC indicating the critical role of HER2 in PEITC-induced apoptosis.

### Overexpression of HER2 enhanced PEITC-induced apoptosis

Next, we were interested to see if HER2 overexpression could enhance PEITC-mediated apoptosis. We transfected MCF-7 cells with HER2-expressing plasmid and treated the cells with PEITC. Using transient transfection, we were able to achieve approximately 2.5-fold overexpression of HER2 in these cells. MCF-7 cells were chosen for HER2 transfection, because MCF-7 cells were relatively resistant to the cytotoxic effects of PEITC (Figure 1A, B). As shown in Figure 4B, overexpression of HER2 led to more PARP cleavage by PEITC treatment. Furthermore, significantly more apoptosis was observed by PEITC treatment in MCF-7 cells overexpressing HER2, as measured by ELISA cell death detection assay, confirming our observation made by western blotting (Additional file 2, Figure S2B).

### Effect of PEITC treatment in cells stably overexpressing HER2

**Figure 5:**
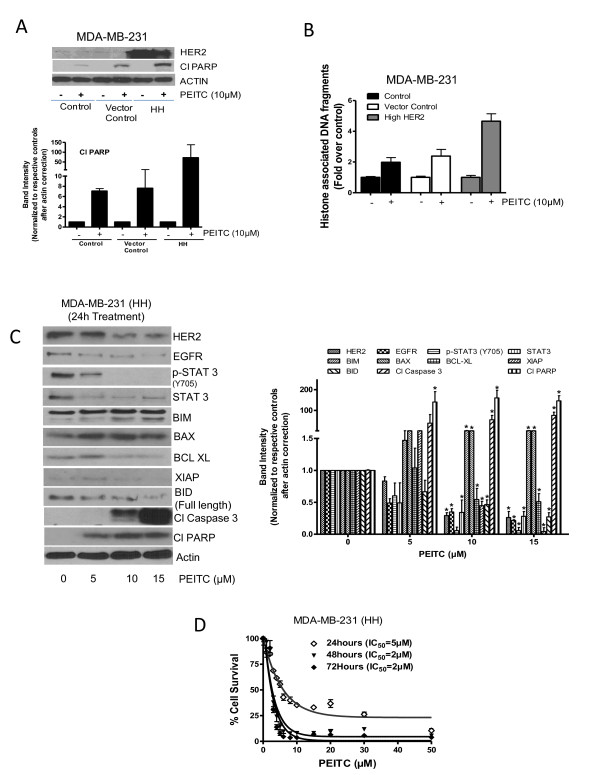
**Effect of phenethyl isothiocyanate (PEITC) on MDA-MB-231 cells expressing high HER2 (HH)**. **(A) **Comparative effect of PEITC treatment on MDA-MB-231 cells with stable overexpression of HER2 relative to parent cells and vector control cells. Cells were treated for 24 h with 10 μM PEITC, and whole lysate was analyzed by western blotting for phosphorylated signal transducer and activator of transcription 3 (p-STAT3) (Y705) and cleavage of poly-ADP ribose polymerase (PARP). **(B) **MDA-MB-231, vector control cells and high HER2 cells were treated with 10 μM of PEITC for 24 h and apoptosis was estimated using ELISA cell death assay by measuring histone associated DNA fragments. **(C) **MDA-MB-231 (HH) cells were treated with different concentrations of PEITC for 24 h, and the whole cell lysates were analyzed by western blotting. **(D) **MDA-MB-231 (HH) cells were treated with PEITC for 24, 48 and 72 h with increasing concentrations of PEITC and cell survival was measured by sulforhodamine B assay. The figures are representative of at least three independent experiments with eight replicates. *Statistically different compared with control (*P *< 0.05).

**Figure 6:**
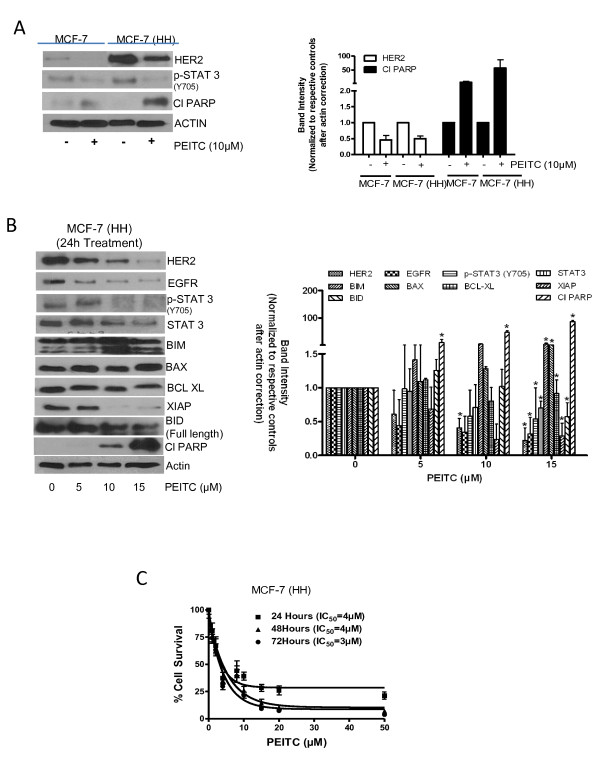
**Effect of phenethyl isothiocyanate (PEITC) on MCF-7 (high HER2 (HH)) cells**. **(A) **Comparative effect of PEITC treatment in MCF-7 cells with stable overexpression of HER2 relative to the parent cells. Cells were treated for 24 h with 10 μM PEITC and whole lysate was analyzed by western blotting for phosphorylated signal transducer and activator of transcription 3 (p-STAT3) (Y705) and cleavage of poly-ADP ribose polymerase (PARP). **(B) **MCF-7 (HH) cells were treated with different concentrations of PEITC for 24 h, and the whole cell lysates were analyzed by western blotting. **(C) **MCF-7 (HH) cells treated with PEITC for 24, 48 and 72 h with increasing concentrations of PEITC and cell survival was measured by sulforhodamine B assay. The figures are representative of at least three independent experiments with eight replicates. *Statistically different compared with control (*P *< 0.05).

Because we observed that transiently overexpressing HER2 enhanced the cytotoxic effects of PEITC, we next sought to determine the effect of PEITC in MDA-MB-231 and MCF-7 cells stably overexpressing HER2 (HH). First of all, we compared the effect of PEITC in both the cell lines stably overexpressing HER2 with that of the cells with constitutive levels. Humongous overexpression of HER2 was observed in stably transfected MDA-MB-231 and MCF-7 cells, respectively, as compared to the parent cell lines or vector transfected control cells (Figures 5A and 6A). In addition increased phosphorylation of STAT3 was observed in both MDA-MB-231 and MCF-7 cells expressing high HER2 levels, indicating the regulation of STAT3 by HER2 (Figures 5A and 6A). Nevertheless, the expression of HER2 was drastically reduced by PEITC treatment in both the cells overexpressing HER2 (Figures 5A and 6A). As shown in Figure 5A, MDA-MB-231 cells overexpressing HER2 (MDA-MB-231 (HH)) showed enhanced PARP cleavage as compared to the parent or vector transfected control cells in response to PEITC treatment. These observations were confirmed by ELISA cell death detection assay, where MDA-MB-231 (HH) cells showed more apoptosis relative to the parent cell line or vector transfected control cells at all the concentrations of PEITC (Figure 5B and Additional file 3, Figure S3A). Similar observations were made in MCF-7 cells overexpressing HER2, but the effect of PEITC was more in MCF-7 (HH) cells as compared to MDA-MB-231 (HH) cells (Figure 6A and Additional file 3, Figure S3B).

To determine the effect of PEITC on HER2 responsive and proapoptotic proteins, HER2-overexpressing cells were treated with varying concentrations of PEITC for 24 h and evaluated by western blot. Consistent with MDA-MB-231 and MCF-7 parent cell lines data, PEITC treatment substantially reduced the expression of HER2, EGFR, STAT3 and phosphorylation of STAT3 in MDA-MB-231 (HH) cells overexpressing HER2 (Figure 5C). Proapoptotic proteins BIM, BAX and BID were highly activated by PEITC treatment, whereas antiapoptotic BCL-XL and XIAP were significantly downregulated (Figure 5C). Substantial cleavage of caspase 3 and PARP was also observed in MDA-MB-231 (HH) cells in response to PEITC treatment (Figure 5C). Notably, cleavage of caspase-3 and/or PARP was substantially more in MDA-MB-231 (HH) cells by PEITC treatment as compared to parental cells. Similar observations were made in MCF-7 (HH) cells overexpressing HER2 (Figure 6B). Next, we wanted to see whether the overexpression of HER2 would increase the cytotoxic potential of PEITC. Therefore a cell survival assay was performed in MDA-MB-231 (HH) and MCF-7 (HH) cells after treatment with PEITC. As shown in Figure 5D, PEITC treatment significantly reduced the survival of MDA-MB-231 (HH) cells with an IC_50 _of 5 μM and 2 μM after 24 h and 72 h of treatment respectively. When compared with parent MDA-MB-231 cells, the IC_50 _of PEITC was about 38% to 50% less in MDA-MB-231 (HH) cells. Similarly, PEITC treatment decreased the survival of MCF-7 (HH) cells with an IC_50 _of 4 μM and 3 μM respectively after 24 h and 72 h treatment (Figure 6C). The IC_50 _of PEITC in MCF-7 (HH) cells was decreased by 50% to 71% as compared to MCF-7 cells by PEITC treatment. Taken together, these results clearly indicated that HER2 makes breast cancer cells more sensitive to the growth suppressive effects of PEITC.

### PEITC decreases mitochondrial membrane potential

**Figure 7:**
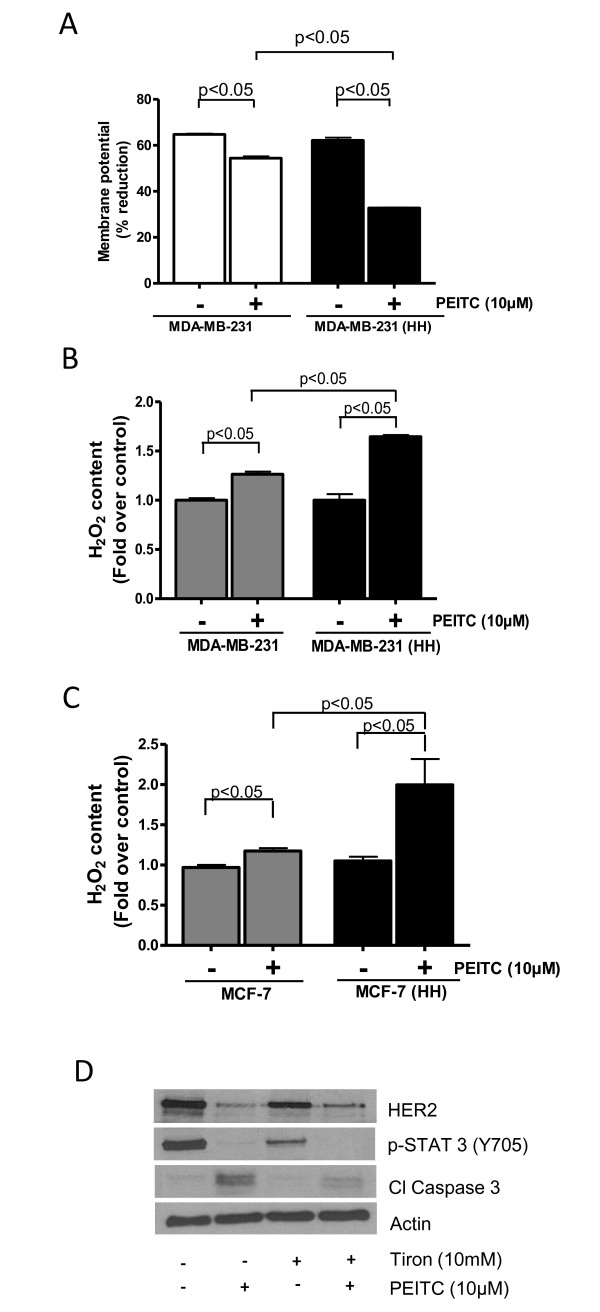
**(PEITC) causes ROS generation and mitochondrial depolarization**. **(A) **MDA-MB-231 and MDA-MB-231 (high HER2 (HH)) cells were treated with PEITC for 24 h and then were labeled with tetramethylrhodamine and analyzed by flow cytometer (n = 3). Hydrogen peroxide content was measured in control or 10 μM PEITC-treated **(B) **MDA-MB-231 and MDA-MB-231 (HH) cells and **(C) **MCF-7 and MCF-7 (HH) cells. **(D) **MDA-MB-231 cells were treated with 10 mM Tiron followed by treatment with 10 μM PEITC for 24 h. The cell lysate was analyzed for HER2 expression. Statistically different when compared with control (*P *< 0.05).

Since we observed the involvement of mitochondrial proteins in PEITC-induced apoptosis, we next determined mitochondrial membrane potential using TMRM as a specific probe. PEITC treatment caused about 15% decrease in the membrane potential in MDA-MB-231 cells as compared to 54% decrease in MDA-MB-231 (HH) (Figure 7A). Once again our results indicated that HER2 sensitizes MDA-MB-231 cells to the mitotoxic effects of PEITC.

### Reactive oxygen species (ROS) induction by PEITC treatment

Depolarization of mitochondria leading to cell death is usually due to excessive generation of ROS in the cells. We therefore measured hydrogen peroxide concentration in the cells using a commercial kit. As shown in Figure 7B, hydrogen peroxide level was much higher in MDA-MB-231 (HH) cells as compared to MDA-MB-231 cells in response to PEITC treatment (Figure 7B). Similar observations were made in MCF-7 (HH) cells (Figure 7C). These results once again indicate that HER2 increased the ability of breast cancer cells to generate hydrogen peroxide in response to PEITC treatment leading to cell death. The next step was to determine whether ROS (hydrogen peroxide) were involved in the downregulation of HER2 and apoptosis by PEITC treatment. In order to prove this association, MDA-MB-231 cells were pretreated with 10 mM Tiron (a general antioxidant) for 1 h to block ROS followed by treatment with 10 μM PEITC for 24 h. As shown in Figure 7D, Tiron treatment considerably blocked the downregulation of HER2 by PEITC treatment. Tiron also substantially decreased the apoptosis induced by PEITC treatment as shown by reduced cleavage of caspase-3 (Figure 7D).

### Tumor therapy model

**Figure 8:**
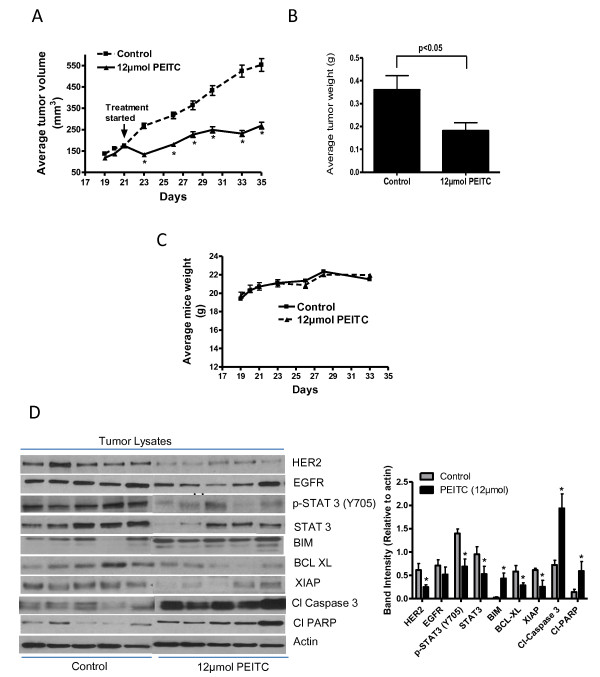
**Phenethyl isothiocyanate (PEITC) suppresses the growth of MDA-MB-231 (high HER2 (HH)) tumors by inhibiting HER2 in SCID/NOD mice**. About 5 × 10^6 ^MDA-MB-231 (HH) cells were subcutaneously implanted into the right flanks of SCID/NOD mice. Once each mouse had a tumor of about 150 mm^3^, mice started receiving 12 μmol of PEITC by oral gavage every day. Tumors were measured three times a week, and each mouse was weighed twice a week. Effect of PEITC on **(A) **tumor volume, **(B) **tumor weight, (C) Mice weight and **(D) **tumor western blots. Tumors were minced, lysed and analyzed for HER2, epidermal growth factor receptor (EGFR), phosphorylated signal transducer and activator of transcription 3 (p-STAT3) (Y-705), STAT3 expression, BIM, B-cell lymphoma-extra large (BCL-XL), X-linked inhibitor of apoptosis protein (XIAP), cleaved poly-ADP ribose polymerase (PARP) and cleaved caspase 3. Blots were stripped and reprobed with actin antibody to verify equal protein loading. Each lane represents a tumor sample from different mice. The blots were quantitated, normalized with actin and represented as bars.

Since we consistently observed that HER2-overexpressing cells were more sensitive to PEITC, we next evaluated the efficacy of PEITC *in vivo*. About 5 × 10^6 ^MDA-MB-231 (HH) cells were implanted in SCID/NOD mice subcutaneously. After each mouse had a tumor of about 150 mm^3^, 12 μmol PEITC treatment by oral gavage started. Our results show that PEITC treatment significantly suppressed the growth of breast tumors (Figure 8A). At day 35 of PEITC treatment, tumor volume in the treated group was reduced by 45% as compared with control group (552.8 ± 90.3 mm^3 ^vs 295.4 ± 44.2 mm^3^; *n *= 10) (Figure 8A). Similarly, the average weight of the tumors dissected from PEITC-treated mice was approximately 43% less than the weight of the tumors from control mice (Figure 8B). The weight of the mice did not change at all during the treatment period, indicating no apparent systemic toxicity in PEITC-treated mice (Figure 8C). The tumors of control and PEITC-treated mice were further analyzed by western blotting and immunofluorescence. Consistent with our *in vitro *results, western blot of the tumor lysate shows that PEITC treatment substantially downregulated HER2 expression and phosphorylation of STAT3 (Figure 8D). We also observed a decrease in EGFR expression in the tumors of PEITC-treated mice, but it was not as pronounced as HER2 downregulation. It is noteworthy that substantial upregulation of BIM and cleavage of caspase 3 and PARP were observed in the tumors of PEITC-treated mice as compared to control indicating apoptosis (Figure 8D). However, expression of antiapoptotic proteins such as BCL-XL and XIAP were reduced (Figure 8D). These observations were confirmed by the immunofluorescence studies in the tumor sections of control and PEITC-treated mice. The staining for HER2 and phosphorylated STAT3 were reduced; whereas cleavage of caspase 3 was increased in the tumor sections from PEITC-treated mice (Additional file 4, Figure S4). Taken together, our results show that tumor growth suppression by PEITC treatment was associated with HER2 downregulation and increased apoptosis, in agreement with our *in vitro *results.

### PEITC enhances the effects of doxorubicin

**Figure 9:**
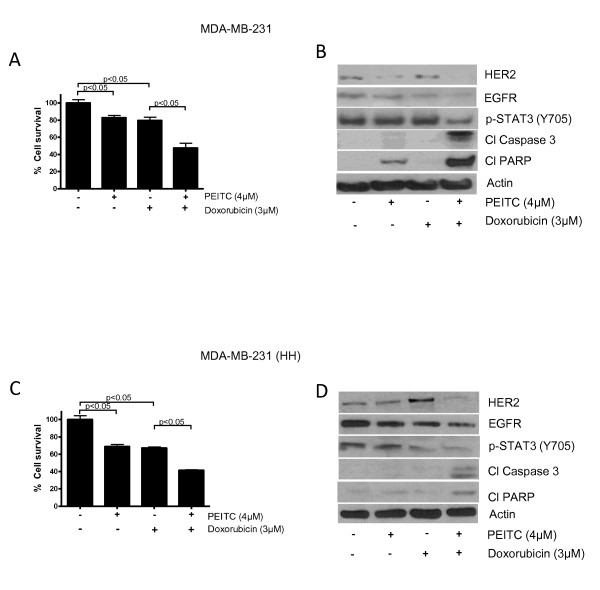
**Phenethyl isothiocyanate (PEITC) enhances the cytotoxic effects of doxorubicin**. **(A) **MDA-MB-231 cells were treated with PEITC (4 μM) and/or doxorubicin (3 μM) for 24 h and analyzed by sulforhodamine B cell survival assay. **(B) **MDA-MB-231 cells were treated with PEITC (4 μM) and/or doxorubicin (3 μM) for 24 h and analyzed by western blotting for HER2 and cleavage of caspase 3 and poly-ADP ribose polymerase (PARP). **(C) **MDA-MB-231 (high HER2 (HH)) cells were treated with 4 μM of PEITC and 3 μM of doxorubicin for 24 h and analyzed by sulforhodamine B cell survival assay. **(D) **The cell lysates from MDA-MB-231 (HH) cells treated with 4 μM of PEITC and/or 3 μM of doxorubicin were analyzed by western blotting for HER2, cleaved caspase 3 and PARP. Actin was used as internal loading control. Statistically different when compared with control (*P *< 0.05).

Next, we wanted to determine whether the effects of doxorubicin could be enhanced at low concentrations, since the regular doses currently being used by the patients are associated with severe cardiotoxicity. Furthermore, HER2 overexpression is known to reduce the efficacy of doxorubicin. In order to do this, cells were treated with sub toxic concentrations of 4 μM PEITC and 3 μM doxorubicin for 24 h. It is important to mention that the IC_50 _values of PEITC and doxorubicin were 8 μM and 6 μM in MDA-MB-231 and 5 μM and 10 μM in MDA-MB-231 (HH) cells after 24 h of treatment. Our results show that individual treatment of 4 μM PEITC or 3 μM doxorubicin reduced the cell survival by 20%. However, combination of PEITC and doxorubicin treatment for 24 h reduced 50% cell survival (Figure 9A). Doxorubicin (3 μM) treatment suppressed HER2 expression modestly, whereas PEITC (4 μM) decreased the expression significantly (Figure 9B). Nonetheless, the combination of PEITC with doxorubicin substantially decreased the expression of HER2 and the phosphorylation of STAT3 in MDA-MB-231 cells (Figure 9B). Consistently, combination treatment showed increased cleavage of caspase 3 and PARP as compared to individual treatment indicating apoptosis (Figure 9B). Similarly in MDA-MB-231 (HH) cells, individual treatment of 4 μM PEITC or 3 μM doxorubicin reduced the survival of cells by about 30%, whereas combination treatment reduced 60% survival (Figure 9C). Modest suppression of HER2 was observed following 24 h of 4 μM PEITC treatment in MDA-MB-231 (HH) cells. However, 3 μM doxorubicin treatment enhanced HER2 expression in these cells. Nevertheless 24 h combination treatment with both the agents completely suppressed the overall expression of HER2 and p-STAT3 (Y705) in these cells (Figure 9D). The enhanced cytotoxic effect of PEITC and doxorubicin combination was also evident by increased cleavage of caspase 3 and PARP (Figure 9D).

## Discussion

Our results indicated that PEITC treatment considerably suppressed the viability of MCF-7 and MDA-MB-231 breast cancer cells. The reduced viability of these cells was associated with HER2/EGFR downregulation. In addition PEITC treatment reduced the expression and the phosphorylation of STAT3. Overexpressing HER2 using HER2 plasmid enhanced the cytotoxic potential of PEITC in these cells. However, silencing HER2 using siRNA blocked the apoptosis induced by PEITC treatment relative to the cells with constitutive levels of HER2. Stable overexpression of HER2 in these cells made the cells more sensitive to the cytotoxic effects of PEITC as indicated by reduced IC_50 _values. Furthermore, our results indicate that PEITC treatment induced hydrogen peroxide production in breast cancer cells leading to mitochondrial membrane depolarization. In line with hydrogen peroxide production and mitochondrial membrane depolarization, PEITC treatment resulted in the release of cytochrome c into the cytosol, leading to the activation of the caspase 3 cascade. Oral administration of PEITC significantly suppressed the growth of breast tumors in SCID/NOD mice. Tumors from PEITC mice demonstrated reduced expression of HER2, EGFR, STAT3, BCL-XL and XIAP, and increased cleavage of BIM, caspase 3 and PARP indicating apoptosis. To the best of our knowledge, our study for the first time implicated the probable role of HER2 in sensitizing breast cancer cells to PEITC-induced apoptosis *in vitro *and *in vivo*.

HER2 is a tyrosine kinase associated with poor prognosis in breast cancer [2]. Substantial evidence indicates its direct role in tumor progression by promoting migration, invasion, antiapoptotic pathways [36] and drug resistance [9]. Under normal conditions HER2 plays an important role in the development of mammary glands and lactation during parturition [37], while deregulation of HER2 leads to the development and progression of breast cancer [38]. Since 1989 efforts have been made to develop therapeutics for HER2-positive breast tumors [39,40]. Trastuzumab (herceptin), a US Food and Drug Administration (FDA)-approved monoclonal antibody against HER2, was apparently effective in the treatment of HER2-positive tumors [41,42]. However, patients with HER2-positive tumors frequently present primary or secondary resistance to this agent [43]. Also, a significant number of patients show moderate to severe toxicity with trastuzumab [44-46]. These detrimental side effects of current targeted therapies raise a need for newer and better therapeutic agents for targeting HER2. Our results show efficacy of PEITC towards HER2-expressing breast cancer cells. Notably, previous studies have shown that PEITC is not toxic to normal cells [47]. Although induction of cell death has been shown by PEITC in MCF-7 cells [48], the effect of PEITC on HER2 has not been reported so far. To ascertain the specificity of PEITC for HER2, we tested the effect of PEITC in two prototype and syngeneic breast cancer cell lines MDA-MB-231, MDA-MB-231 (HH), MCF-7 and MCF-7 (HH), which have varying levels of HER2. Interestingly we observed increased sensitivity of the cells with higher levels of HER2 towards PEITC. Some previous studies have shown reduced efficiency of chemotherapeutic drugs due to HER2 overexpression [13].

HER2 functions through homodimerization and heterodimerization with other EGFR family receptors to activate further downstream effectors [12]. Some studies indicate the role of STAT3 in EGFR signaling in various cancers [49,50]. Our current study also indicated EGFR inhibition in breast cancer cells by PEITC treatment. Furthermore, inhibition of STAT3 expression and phosphorylation by PEITC treatment in our model indicated the inhibition of survival pathways in breast cancer cells. In agreement with our studies, PEITC has been shown to suppress STAT3 activation in prostate cancer cells [51]. Silencing and overexpression of HER2 showed changes in STAT3 phosphorylation. Though there is no established correlation yet between HER2 and STAT3, our results indicated that HER2 might be regulating STAT3. Detailed studies are needed to further confirm and establish a relationship between HER2 and STAT3.

During the intrinsic mitochondrial death pathway, cytochrome c is released from the mitochondria into the cytosol due to a decrease in mitochondrial membrane potential [52]. Mitochondrial membrane potential is compromised as a result of ROS generation. Our results show that PEITC treatment caused ROS generation and mitochondrial depolarization, resulting in the release of cytochrome c, hence activating caspase 3 mediated apoptosis in agreement with the outcome of other study [53]. Antioxidant Tiron significantly blocked the decrease in HER2 expression and apoptosis by PEITC treatment suggesting the regulation of HER2 by ROS. A recent study suggested that certain genes that are upregulated in HER2 tumors were activated during oxidative stress [48]. Interestingly, the extent of ROS generation and mitochondrial depolarization by PEITC was much higher in HER2 overexpressing cells as compared to parent cells. These observations suggest a link between ROS, HER2 and mitochondrial damage by PEITC in breast cancer cells. Nonetheless, further studies are needed to correlate HER2 and mitochondrial functions. A recent study indicated the involvement of mitochondrial STAT3 in inhibiting ROS production via mitochondrial electron transport chain (ETC) complex I [54]. We also observed inhibition of mitochondrial STAT3 by PEITC treatment in breast cancer cells (data not shown). It was not clear at this point whether ROS generation by PEITC in our model was through the interaction of mitochondrial STAT3 with ETC complex I and needs further investigation. In our studies we also observed significant inhibition of XIAP by PEITC treatment. XIAP is a known inhibitor of apoptosis [55], and transmits survival signals in breast cancer cells [56]. Second mitochondria-derived activator of caspases (Smac) is a known repressor of XIAP and is elevated during apoptosis. We did not observe any significant change in the expression of Smac by PEITC treatment indicating Smac-independent suppression of XIAP in our model.

Oral administration of 12 μmol PEITC significantly suppressed the growth of breast tumor *in vivo*. In a recent study oral administration of 10 μmol/kg PEITC in rats resulted in the peak plasma concentration of 9.2 ± 0.6 μM PEITC [57]. Notably, the IC_50 _of PEITC in HER2 overexpressing cells in our model was less than 5 μM. These results indicate that the therapeutic concentration of PEITC can be achieved clinically in human patients.

Doxorubicin has been used for breast cancer treatment for a long time, but its use has been associated with dose-related acute and chronic toxicity in most of the patients. Several measures have been taken to reduce its toxicity, such as combining it with other chemotherapeutic agents to reduce its dose without compromising its efficacy. Interestingly, our results showed that the cytotoxic effects of doxorubicin can be enhanced by concomitant use of PEITC. Combination of doxorubicin with PEITC effectively suppressed HER2 and STAT3 phosphorylation in breast cancer cells, resulting in enhanced apoptosis as compared to individual treatments respectively. These observations have clinical relevance as our data suggests that the effect of doxorubicin at low doses can be increased by PEITC in HER2-expressing breast cancer cells, which show poor response to doxorubicin therapy in general. Detailed *in vitro *and *in vivo *studies are required to establish this association.

Taken together, our results indicated that (i) HER2 is a potential molecular target of PEITC in breast cancer cells *in vitro *and *in vivo *and (ii) PEITC has a potential to enhance the cytotoxic effects of doxorubicin. Our study suggests a unique specificity of PEITC towards HER2-overexpressing breast cancer cells, indicating that PEITC could be beneficial to a subset of patient population overexpressing HER2.

## Conclusions

Our studies clearly indicate a unique specificity of PEITC towards HER2 overexpressing breast cancer cells, indicating that PEITC could be beneficial to a subset of patient population overexpressing HER2, alone or in combination with doxorubicin.

## Competing interests

The authors declare that they have no competing interests.

## Authors' contributions

PG was responsible for designing the study, performing the experiments and writing the first draft of the manuscript. SKS was responsible for designing the study, analyzing the data and writing the manuscript. All authors read an approved the final manuscript.

## Pre-publication history

The pre-publication history for this paper can be accessed here:

http://www.biomedcentral.com/1741-7015/10/80/prepub

## Supplementary Material

Additional file 1 **Figure S1**. Phenethyl isothiocyanate (PEITC) induces histone associated fragmentation in breast cancer cells. Apoptosis induction was measured by enzyme-linked immunosorbent assay (ELISA) cell death detection method in **(A) **MDA-MB-231 and **(B) **MCF-7 (n = 3). Each experiment was repeated more than three times independently. *Statistically different when compared with control (*P *< 0.05). Click here for file

Additional file 2 **Figure S2**. Change in HER2 expression modulates the effect of phenethyl isothiocyanate (PEITC). **(A) **Effect of PEITC in HER2-silenced MDA-MB-231 cells. At 48 h after transfection of cells with HER2 siRNA, cells were treated with or without 10 μM PEITC for 24 h. Apoptosis was measured by enzyme-linked immunosorbent assay (ELISA) cell death detection method after silencing HER2 in MDA-MB-231 (n = 15). **(B) **Effect of HER2 overexpression on apoptosis induction in MCF-7 by PEITC treatment. After 48 h of HER2 transfection, cells were treated with or without 10 μM PEITC for 24 h. The means of three independent experiments performed in triplicate are shown. The induction of apoptosis by ELISA cell death detection method in HER2 overexpressing MCF-7 cells (n = 3). Click here for file

Additional file 3 **Figure S3**. Change in HER2 expression modulates the effect of phenethyl isothiocyanate (PEITC). **(A) **Comparative effect of PEITC treatment on MDA-MB-231 cells with stable overexpression of HER2 relative to parent cells. Cells were treated for 24 h with 10 μM PEITC and apoptosis measured by enzyme-linked immunosorbent assay (ELISA) cell death detection method in MDA-MB-231 parent cells and the cells with stable overexpression of HER2, after treatment with 10 μM PEITC for 24 h. **(B) **Comparative effect of PEITC treatment in MCF-7 cells with stable overexpression of HER2 relative to the parent cells. Cells were treated for 24 h with 10 μM PEITC and apoptosis measured by ELISA cell death detection method in MCF-7 parent cells and the cells with stable overexpression of HER2, after treatment with PEITC (10 μM) for 24 h. The figures are representative of at least three independent experiments with eight replicates. *Statistically different compared with control (*P *< 0.05). Click here for file

Additional file 4 **Figure S4**. Phenethyl isothiocyanate (PEITC) suppresses the growth of MDA-MB-231 (high HER2 (HH)) tumors by inhibiting HER2 in SCID/NOD mice. About 5 × 10^6 ^MDA-MB-231 (HH) cells were subcutaneously implanted into the right flanks of SCID/NOD mice. Once each mouse had a tumor of about 150 mm^3^, mice started receiving 12 μmol of PEITC by oral gavage every day. About 20 micron sections were obtained from snap frozen tumor tissues for tumor analysis. Immunofluorescence for HER2, phosphorylated signal transducer and activator of transcription 3 (p-STAT3) (Y-705) and cleaved caspase 3 in tumor sections from control and PEITC treated mice. The red staining represents the expression of HER2, p-STAT3 (Y705) and cleaved caspase 3. Click here for file

## References

[B1] Harris L Fritsche H Mennel R Norton L Ravdin P Taube S Somerfield MR Hayes DF Bast RC Jr American Society of Clinical Oncology 2007 update of recommendations for the use of tumor markers in breast cancer J Clin Oncol 2007 25 5287 5312 10.1200/JCO.2007.14.2364 17954709

[B2] Slamon DJ Clark GM Wong SG Levin WJ Ullrich A McGuire WL Human breast cancer: correlation of relapse and survival with amplification of the HER-2/neu oncogene Science 1987 235 177 182 10.1126/science.3798106 3798106

[B3] King CR Kraus MH Aaronson SA Amplification of a novel v-erbB-related gene in a human mammary carcinoma Science 1985 229 974 976 10.1126/science.2992089 2992089

[B4] Yokota J Yamamoto T Toyoshima K Terada M Sugimura T Battifora H Cline MJ Amplification of c-erbB-2 oncogene in human adenocarcinomas in vivo Lancet 1986 1 765 767 2870269 10.1016/s0140-6736(86)91782-4

[B5] Semba K Kamata N Toyoshima K Yamamoto T A v-erbB-related protooncogene, c-erbB-2, is distinct from the c-erbB-1/epidermal growth factor-receptor gene and is amplified in a human salivary gland adenocarcinoma Proc Natl Acad Sci USA 1985 82 6497 6501 10.1073/pnas.82.19.6497 2995967 PMC390744

[B6] Fukushige S Matsubara K Yoshida M Sasaki M Suzuki T Semba K Toyoshima K Yamamoto T Localization of a novel v-erbB-related gene, c-erbB-2, on human chromosome 17 and its amplification in a gastric cancer cell line Mol Cell Biol 1986 6 955 958 2430175 10.1128/mcb.6.3.955-958.1986 PMC367597

[B7] Lee J Dull TJ Lax I Schlessinger J Ullrich A HER2 cytoplasmic domain generates normal mitogenic and transforming signals in a chimeric receptor EMBO J 1989 8 167 173 2565808 10.1002/j.1460-2075.1989.tb03361.x PMC400786

[B8] Ekerljung L Lindborg M Gedda L Frejd FY Carlsson J Lennartsson J Dimeric HER2-specific affibody molecules inhibit proliferation of the SKBR-3 breast cancer cell line Biochem Biophys Res Commun 2008 377 489 494 10.1016/j.bbrc.2008.10.027 18930032

[B9] Boone JJ Bhosle J Tilby MJ Hartley JA Hochhauser D Involvement of the HER2 pathway in repair of DNA damage produced by chemotherapeutic agents Mol Cancer Ther 2009 8 3015 3023 10.1158/1535-7163.MCT-09-0219 19887555

[B10] Kumar R Yarmand-Bagheri R The role of HER2 in angiogenesis Semin Oncol 2001 28 Suppl 16 27 32 11706393 10.1016/s0093-7754(01)90279-9

[B11] Palmieri D Bronder JL Herring JM Yoneda T Weil RJ Stark AM Kurek R Vega-Valle E Feigenbaum L Halverson D Vortmeyer AO Steinberg SM Aldape K Steeg PS Her-2 overexpression increases the metastatic outgrowth of breast cancer cells in the brain Cancer Res 2007 67 4190 4198 10.1158/0008-5472.CAN-06-3316 17483330

[B12] Doroshow JH Doxorubicin-induced cardiac toxicity New Engl J Med 1991 324 843 845 10.1056/NEJM199103213241210 1997858

[B13] Shi Y Moon M Dawood S McManus B Liu PP Mechanisms and management of doxorubicin cardiotoxicity Herz 2011 36 296 305 10.1007/s00059-011-3470-3 21656050

[B14] Horie T Ono K Nishi H Nagao K Kinoshita M Watanabe S Kuwabara Y Nakashima Y Takanabe-Mori R Nishi E Hasegawa K Kita T Kimura T Acute doxorubicin cardiotoxicity is associated with miR-146a-induced inhibition of the neuregulin-ErbB pathway Cardiovasc Res 2010 87 656 664 10.1093/cvr/cvq148 20495188 PMC2920811

[B15] Pritchard KI Shepherd LE O'Malley FP Andrulis IL Tu D Bramwell VH Levine MN HER2 and responsiveness of breast cancer to adjuvant chemotherapy N Engl J Med 2006 354 2103 2111 10.1056/NEJMoa054504 16707747

[B16] Hayes DF Thor AD Dressler LG Weaver D Edgerton S Cowan D Broadwater G Goldstein LJ Martino S Ingle JN Henderson IC Norton L Winer EP Hudis CA Ellis MJ Berry DA Cancer and Leukemia Group B (CALGB) Investigators HER2 and response to paclitaxel in node-positive breast cancer N Engl J Med 2007 357 1496 1506 10.1056/NEJMoa071167 17928597

[B17] Strohecker AM Yehiely F Chen F Cryns VL Caspase cleavage of HER-2 releases a Bad-like cell death effector J Biol Chem 2008 283 18269 18282 10.1074/jbc.M802156200 18420586 PMC2440621

[B18] Boggs DA Palmer JR Wise LA Spiegelman D Stampfer MJ Adams-Campbell LL Rosenberg L Fruit and vegetable intake in relation to risk of breast cancer in the Black Women's Health Study Am J Epidemiol 2010 172 1268 1279 10.1093/aje/kwq293 20937636 PMC3025632

[B19] Ambrosone CB McCann SE Freudenheim JL Marshall JR Zhang Y Shields PG Breast cancer risk in premenopausal women is inversely associated with consumption of broccoli, a source of isothiocyanates, but is not modified by GST genotype J Nutr 2004 134 1134 1138 15113959 10.1093/jn/134.5.1134

[B20] Wattenberg LW Inhibition of carcinogenesis by minor anutrient constituents of the diet Proc Nutr Soc 1990 49 173 183 10.1079/PNS19900022 2236085

[B21] Palmer S Diet, nutrition, and cancer Prog Food Nutr Sci 1985 9 283 341 3010379

[B22] Fowke JH Chung FL Jin F Qi D Cai Q Conaway C Cheng JR Shu XO Gao YT Zheng W Urinary isothiocyanate levels, brassica, and human breast cancer Cancer Res 2003 63 3980 3986 12873994

[B23] Wang X Di Pasqua AJ Govind S McCracken E Hong C Mi L Mao Y Wu JY Tomita Y Woodrick JC Fine RL Chung FL Selective depletion of mutant p53 by cancer chemopreventive isothiocyanates and their structure-activity relationships J Med Chem in press 10.1021/jm101199t PMC3139710 21241062

[B24] Kelloff GJ Crowell JA Hawk ET Steele VE Lubet RA Boone CW Covey JM Doody LA Omenn GS Greenwald P Hong WK Parkinson DR Bagheri D Baxter GT Blunden M Doeltz MK Eisenhauer KM Johnson K Knapp GG Longfellow DG Malone WF Nayfield SG Seifried HE Swall LM Sigman CC Strategy and planning for chemopreventive drug development: clinical development plans II J Cell Biochem Suppl 1996 26 54 71 9154168 10.1002/jcb.240630705

[B25] Sahu RP Batra S Kandala PK Brown TL Srivastava SK The role of K-ras gene mutation in TRAIL-induced apoptosis in pancreatic and lung cancer cell lines Cancer Chemother Pharmacol 2011 67 481 487 10.1007/s00280-010-1463-1 20848283 PMC5842667

[B26] Sahu RP Srivastava SK The role of STAT-3 in the induction of apoptosis in pancreatic cancer cells by benzyl isothiocyanate J Natl Cancer Inst 2009 101 176 193 10.1093/jnci/djn470 19176463 PMC2724856

[B27] Zhang R Humphreys I Sahu RP Shi Y Srivastava SK In vitro and in vivo induction of apoptosis by capsaicin in pancreatic cancer cells is mediated through ROS generation and mitochondrial death pathway Apoptosis 2008 13 1465 1478 10.1007/s10495-008-0278-6 19002586

[B28] Sahu RP Zhang R Batra S Shi Y Srivastava SK Benzyl isothiocyanate-mediated generation of reactive oxygen species causes cell cycle arrest and induces apoptosis via activation of MAPK in human pancreatic cancer cells Carcinogenesis 2009 30 1744 1753 10.1093/carcin/bgp157 19549704 PMC2757546

[B29] Kandala PK Srivastava SK Regulation of macroautophagy in ovarian cancer cells in vitro and in vivo by controlling glucose regulatory protein 78 and AMPK Oncotarget 2012 3 435 449 22564965 10.18632/oncotarget.483 PMC3380578

[B30] Boreddy SR Pramanik KC Srivastava SK Pancreatic tumor suppression by benzyl isothiocyanate is associated with inhibition of PI3K/AKT/FOXO pathway Clin Cancer Res 2011 17 1784 1795 10.1158/1078-0432.CCR-10-1891 21350002 PMC3076680

[B31] Euhus DM Hudd C LaRegina MC Johnson FE Tumor measurement in the nude mouse Journal of Surgical Oncology 1986 31 229 234 10.1002/jso.2930310402 3724177

[B32] Siddiqa A Long LM Li L Marciniak RA Kazhdan I Expression of HER-2 in MCF-7 breast cancer cells modulates anti-apoptotic proteins Survivin and Bcl-2 via the extracellular signal-related kinase (ERK) and phosphoinositide-3 kinase (PI3K) signalling pathways BMC Cancer 2008 8 129 10.1186/1471-2407-8-129 18454859 PMC2386479

[B33] Arora P Cuevas BD Russo A Johnson GL Trejo J Persistent transactivation of EGFR and ErbB2/HER2 by protease-activated receptor-1 promotes breast carcinoma cell invasion Oncogene 2008 27 4434 4445 10.1038/onc.2008.84 18372913 PMC2874884

[B34] Asada S Choi Y Yamada M Wang SC Hung MC Qin J Uesugi M External control of Her2 expression and cancer cell growth by targeting a Ras-linked coactivator Proc Natl Acad Sci USA 2002 99 12747 12752 10.1073/pnas.202162199 12242338 PMC130531

[B35] Garcia R Bowman TL Niu G Yu H Minton S Muro-Cacho CA Cox CE Falcone R Fairclough R Parsons S Laudano A Gazit A Levitzki A Kraker A Jove R Constitutive activation of Stat3 by the Src and JAK tyrosine kinases participates in growth regulation of human breast carcinoma cells Oncogene 2001 20 2499 2513 10.1038/sj.onc.1204349 11420660

[B36] Hynes NE Lane HA ERBB receptors and cancer: the complexity of targeted inhibitors Nat Rev Cancer 2005 5 341 354 10.1038/nrc1609 15864276

[B37] Jones FE Stern DF Expression of dominant-negative ErbB2 in the mammary gland of transgenic mice reveals a role in lobuloalveolar development and lactation Oncogene 1999 18 3481 3490 10.1038/sj.onc.1202698 10376526

[B38] Hynes NE Stern DF The biology of erbB-2/neu/HER-2 and its role in cancer Biochim Biophys Acta 1994 1198 165 184 7819273 10.1016/0304-419x(94)90012-4

[B39] Shepard HM Lewis GD Sarup JC Fendly BM Maneval D Mordenti J Figari I Kotts CE Palladino MA Jr Ullrich A Slamon D Monoclonal antibody therapy of human cancer: taking the HER2 protooncogene to the clinic J Clin Immunol 1991 11 117 127 10.1007/BF00918679 1679763

[B40] Hudziak RM Lewis GD Winget M Fendly BM Shepard HM Ullrich A p185HER2 monoclonal antibody has antiproliferative effects in vitro and sensitizes human breast tumor cells to tumor necrosis factor Mol Cell Biol 1989 9 1165 1172 2566907 10.1128/mcb.9.3.1165-1172.1989 PMC362707

[B41] Goldenberg MM Trastuzumab, a recombinant DNA-derived humanized monoclonal antibody, a novel agent for the treatment of metastatic breast cancer Clin Ther 1999 21 309 318 10.1016/S0149-2918(00)88288-0 10211534

[B42] Graziano C HER-2 breast assay, linked to Herceptin, wins FDA's okay CAP Today 1998 12 1, 14 16 10187049

[B43] Wilken JA Maihle NJ Primary trastuzumab resistance: new tricks for an old drug Ann NY Acad Sci 2010 1210 53 65 10.1111/j.1749-6632.2010.05782.x 20973799 PMC3045786

[B44] Abulkhair O El Melouk W Delayed Paclitaxel-trastuzumab-induced interstitial pneumonitis in breast cancer Case Rep Oncol 2011 4 186 191 10.1159/000326063 21516267 PMC3080783

[B45] Tanz R Mahfoud T Bazine A Khmamouch R Bensouda Y Ismaili N Benjaafar N El Gueddari BK Ichou M Errihani H Cardiac safety of trastuzumab in adjuvant: a review across 53 observations [in French] J Gynecol Obstet Biol Reprod (Paris) 2011 40 144 148 10.1016/j.jgyn.2010.12.003 21227599

[B46] Chien KR Myocyte survival pathways and cardiomyopathy: implications for trastuzumab cardiotoxicity Semin Oncol 2000 27 Suppl 11 9 14 11236034

[B47] Wu XJ Hua X Targeting ROS: selective killing of cancer cells by a cruciferous vegetable derived pro-oxidant compound Cancer Biol Ther 2007 6 646 647 10.4161/cbt.6.5.4092 17387274

[B48] Toullec A Gerald D Despouy G Bourachot B Cardon M Lefort S Richardson M Rigaill G Parrini MC Lucchesi C Bellanger D Stern MH Dubois T Sastre-Garau X Delattre O Vincent-Salomon A Mechta-Grigoriou F Oxidative stress promotes myofibroblast differentiation and tumour spreading EMBO Mol Med 2010 2 211 230 10.1002/emmm.201000073 20535745 PMC3377319

[B49] Takata S Takigawa N Segawa Y Kubo T Ohashi K Kozuki T Teramoto N Yamashita M Toyooka S Tanimoto M Kiura K STAT3 expression in activating EGFR-driven adenocarcinoma of the lung Lung Cancer 2012 75 24 29 10.1016/j.lungcan.2011.05.015 21684622

[B50] Zhou W Grandis JR Wells A STAT3 is required but not sufficient for EGF receptor-mediated migration and invasion of human prostate carcinoma cell lines Br J Cancer 2006 95 164 171 10.1038/sj.bjc.6603234 16804520 PMC2360627

[B51] Gong A He M Krishna Vanaja D Yin P Karnes RJ Young CY Phenethyl isothiocyanate inhibits STAT3 activation in prostate cancer cells Mol Nutr Food Res 2009 53 878 886 10.1002/mnfr.200800253 19437484 PMC3964815

[B52] La Piana G Fransvea E Marzulli D Lofrumento NE Mitochondrial membrane potential supported by exogenous cytochrome c oxidation mimics the early stages of apoptosis Biochem Biophys Res Commun 1998 246 556 561 10.1006/bbrc.1998.8664 9610401

[B53] Syed Alwi SS Cavell BE Donlevy A Packham G Differential induction of apoptosis in human breast cancer cell lines by phenethyl isothiocyanate, a glutathione depleting agent Cell Stress Chaperones in press 10.1007/s12192-012-0329-3 PMC3535168 22351438

[B54] Szczepanek K Chen Q Larner AC Lesnefsky EJ Cytoprotection by the modulation of mitochondrial electron transport chain: the emerging role of mitochondrial STAT3 Mitochondrion 2012 12 180 189 10.1016/j.mito.2011.08.011 21930250 PMC3278553

[B55] Deveraux QL Takahashi R Salvesen GS Reed JC X-linked IAP is a direct inhibitor of cell-death proteases Nature 1997 388 300 304 10.1038/40901 9230442

[B56] Dubrez-Daloz L Dupoux A Cartier J IAPs: more than just inhibitors of apoptosis proteins Cell Cycle 2008 7 1036 1046 10.4161/cc.7.8.5783 18414036

[B57] Ji Y Kuo Y Morris ME Pharmacokinetics of dietary phenethyl isothiocyanate in rats Pharm Res 2005 22 1658 1666 10.1007/s11095-005-7097-z 16180123

