# Supplementary material for: Antitumor activity of phenethyl isothiocyanate in HER2-positive breast cancer models
Source: BMC Med. 2012 Jul 24;10:80. doi: 10.1186/1741-7015-10-80 (PMC3412708; doi:10.1186/1741-7015-10-80)
Supplement: Additional file 1 — Figure S1. Phenethyl isothiocyanate (PEITC) induces histone associated fragmentation in breast cancer cells. Apoptosis induction was measured by enzyme-linked immunosorbent assay (ELISA) cell death detection method in (A) MDA-MB-231 and (B) MCF-7 (n = 3). Each experiment was repeated more than three times independently. *Statistically different when compared with control (P < 0.05). [file 1741-7015-10-80-S1.PDF]

# Figure S1

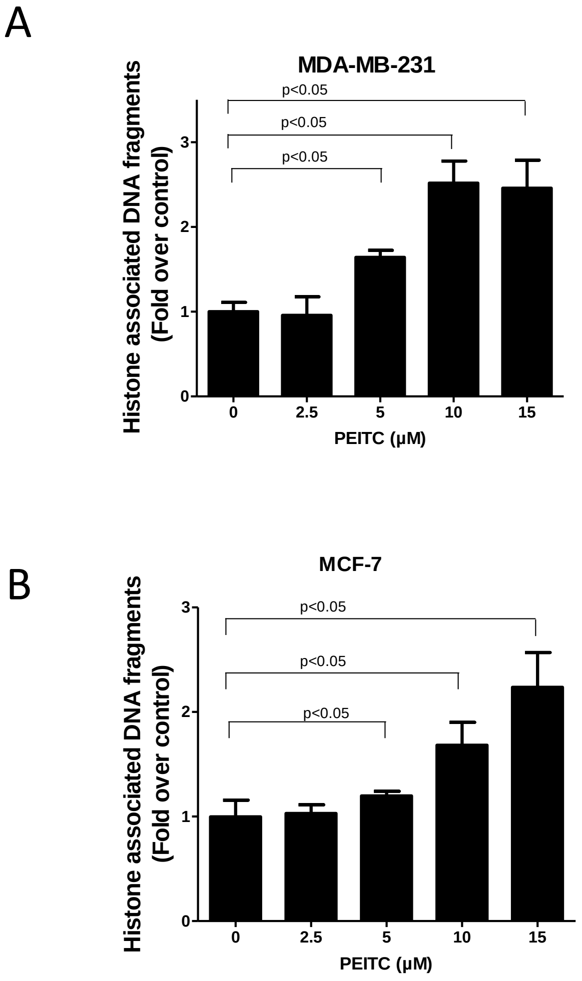


**Figure S1: PEITC induces histone associated fragmentation in breast cancer cells.** Apoptosis induction was measured by ELISA cell death detection method in (A) MDA-MB-231 and (B) MCF-7 (n=3). Each experiment was repeated more than three times independently. *Statistically different when compared with control ($p<0.05$).
