# Supplementary material for: Antitumor activity of phenethyl isothiocyanate in HER2-positive breast cancer models
Source: BMC Med. 2012 Jul 24;10:80. doi: 10.1186/1741-7015-10-80 (PMC3412708; doi:10.1186/1741-7015-10-80)
Supplement: Additional file 2 — Figure S2. Change in HER2 expression modulates the effect of phenethyl isothiocyanate (PEITC). (A) Effect of PEITC in HER2-silenced MDA-MB-231 cells. At 48 h after transfection of cells with HER2 siRNA, cells were treated with or without 10 μM PEITC for 24 h. Apoptosis was measured by enzyme-linked immunosorbent assay (ELISA) cell death detection method after silencing HER2 in MDA-MB-231 (n = 15). (B) Effect of HER2 overexpression on apoptosis induction in MCF-7 by PEITC treatment. After 48 h of HER2 transfection, cells were treated with or without 10 μM PEITC for 24 h. The means of three independent experiments performed in triplicate are shown. The induction of apoptosis by ELISA cell death detection method in HER2 overexpressing MCF-7 cells (n = 3). [file 1741-7015-10-80-S2.PDF]

Figure S2

A

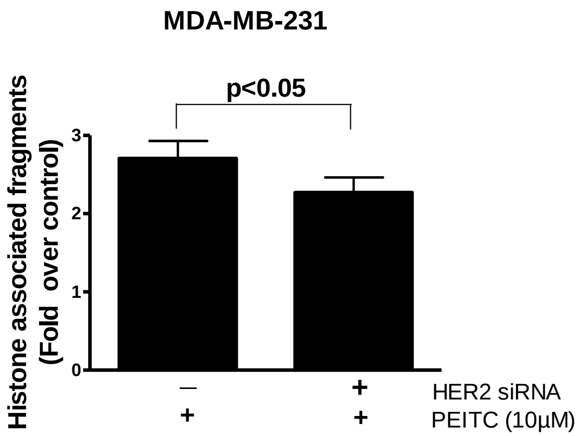

B

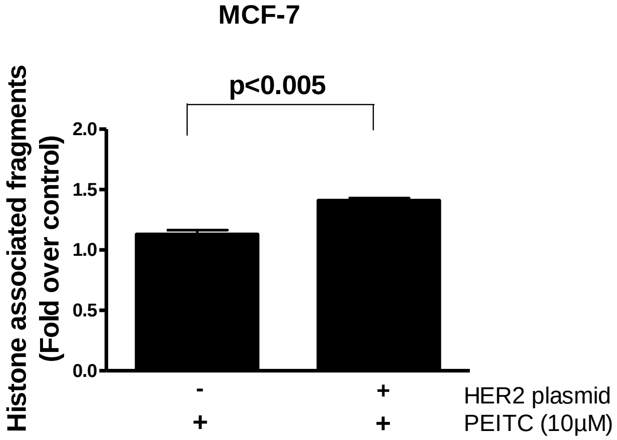

Figure S2: Change in HER2 expression modulates the effect of PEITC. (A) Effect of PEITC in HER2 silenced MDA-MB-231 cells. Forty eight hour after transfection of cells with HER2 siRNA, cells were treated with or without 10µM PEITC for 24h. Apoptosis was measured by ELISA cell death detection method after silencing HER2 in MDA-MB-231 (n=15). (B) Effect of HER2 overexpression on apoptosis induction in MCF-7 by PEITC treatment. After 48 hours of HER2 transfection, cells were treated with or without 10µM PEITC for 24 hours. Means of three independent experiments performed in triplicate are shown. The induction of apoptosis by ELISA cell death detection method in HER2 overexpressing MCF-7 cells (n=3).
