# Supplementary material for: Antitumor activity of phenethyl isothiocyanate in HER2-positive breast cancer models
Source: BMC Med. 2012 Jul 24;10:80. doi: 10.1186/1741-7015-10-80 (PMC3412708; doi:10.1186/1741-7015-10-80)
Supplement: Additional file 3 — Figure S3. Change in HER2 expression modulates the effect of phenethyl isothiocyanate (PEITC). (A) Comparative effect of PEITC treatment on MDA-MB-231 cells with stable overexpression of HER2 relative to parent cells. Cells were treated for 24 h with 10 μM PEITC and apoptosis measured by enzyme-linked immunosorbent assay (ELISA) cell death detection method in MDA-MB-231 parent cells and the cells with stable overexpression of HER2, after treatment with 10 μM PEITC for 24 h. (B) Comparative effect of PEITC treatment in MCF-7 cells with stable overexpression of HER2 relative to the parent cells. Cells were treated for 24 h with 10 μM PEITC and apoptosis measured by ELISA cell death detection method in MCF-7 parent cells and the cells with stable overexpression of HER2, after treatment with PEITC (10 μM) for 24 h. The figures are representative of at least three independent experiments with eight replicates. *Statistically different compared with control (P < 0.05). [file 1741-7015-10-80-S3.PDF]

Figure S3

A

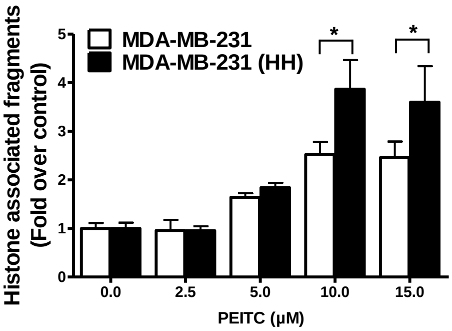

B

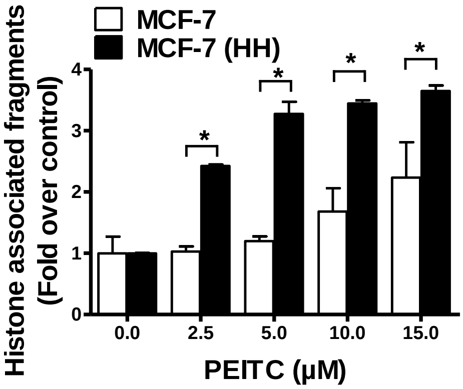

Figure S3: Change in HER2 expression modulates the effect of PEITC. (A) Comparative effect of PEITC treatment on MDA-MB-231 cells with stable overexpression of HER2 relative to parent cells. Cells were treated for 24 h with 10µM PEITC and apoptosis measured by ELISA cell death detection method in MDA-MB-231 parent cells and the cells with stable overexpression of HER2, after treatment with 10µM PEITC for 24 h. (B) Comparative effect of PEITC treatment in MCF-7 cells with stable overexpression of HER2 relative to the parent cells. Cells were treated for 24 h with 10µM PEITC and apoptosis measured by ELISA cell death detection method in MCF-7 parent cells and the cells with stable overexpression of HER2, after treatment with PEITC (10µM) for 24 h. The figures are representative of at least three independent experiments with 8 replicates. *Statistically different compared with control ($p<0.05$).
