# Supplementary material for: Antitumor activity of phenethyl isothiocyanate in HER2-positive breast cancer models
Source: BMC Med. 2012 Jul 24;10:80. doi: 10.1186/1741-7015-10-80 (PMC3412708; doi:10.1186/1741-7015-10-80)
Supplement: Additional file 4 — Figure S4. Phenethyl isothiocyanate (PEITC) suppresses the growth of MDA-MB-231 (high HER2 (HH)) tumors by inhibiting HER2 in SCID/NOD mice. About 5 × 10^6^ MDA-MB-231 (HH) cells were subcutaneously implanted into the right flanks of SCID/NOD mice. Once each mouse had a tumor of about 150 mm3, mice started receiving 12 μmol of PEITC by oral gavage every day. About 20 micron sections were obtained from snap frozen tumor tissues for tumor analysis. Immunofluorescence for HER2, phosphorylated signal transducer and activator of transcription 3 (p-STAT3) (Y-705) and cleaved caspase 3 in tumor sections from control and PEITC treated mice. The red staining represents the expression of HER2, p-STAT3 (Y705) and cleaved caspase 3. [file 1741-7015-10-80-S4.PDF]

Figure S4

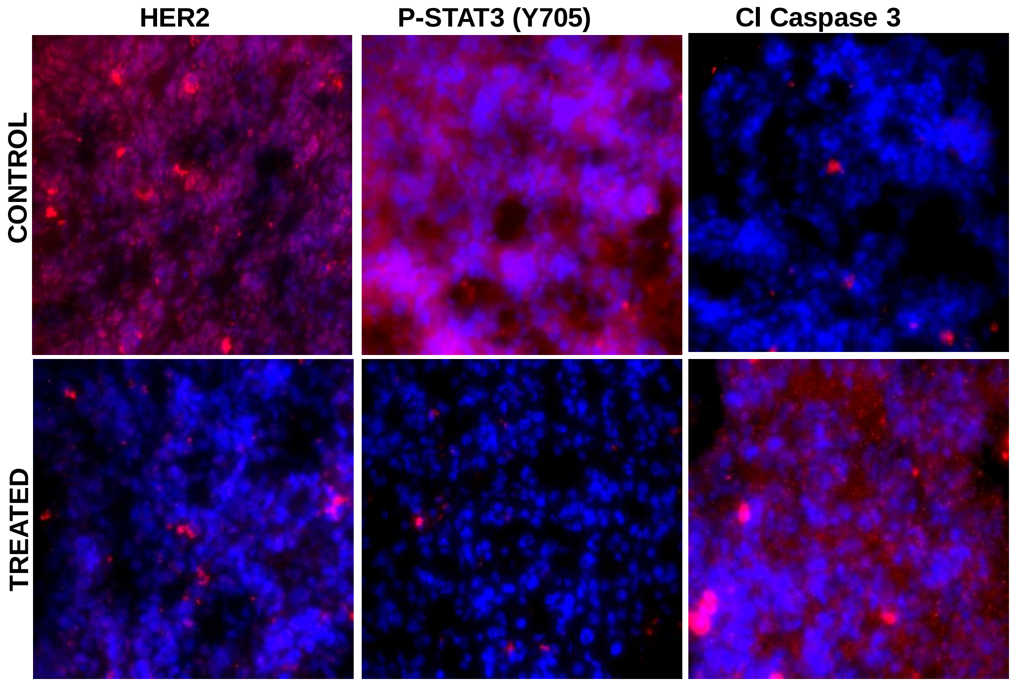

Figure S4: PEITC suppresses the growth of MDA-MB-231 (HH) tumors by inhibiting HER2 in SCID/NOD mice. About $5X10^6$ MDA-MB-231 (HH) cells were subcutaneously implanted into the right flanks of SCID/NOD mice. Once each mouse had a tumor of about $150mm^3$, mice started receiving 12µmol of PEITC by oral gavage every day. About 20µm sections were obtained from snap frozen tumor tissues for tumor analysis. Immunofluorescence for HER2, p-STAT3 (Y-705) and cleaved caspase 3 in tumor sections from control and PEITC treated mice. The red staining represents the expression of HER2, p-STAT3 (Y705) and cleaved caspase 3.
